# Relational Spirituality and Transgenerational Obligations: The Role of Family in Lay Explanatory Models of Post-traumatic Stress Disorder in Male Cameroonian Asylum Seekers and Undocumented Migrants in Europe

**DOI:** 10.3389/fpsyt.2021.621918

**Published:** 2021-04-20

**Authors:** Freyja Grupp, Sara Skandrani, Marie Rose Moro, Ricarda Mewes

**Affiliations:** ^1^Division of Clinical Psychology, Department of Psychology, University of Marburg, Marburg, Germany; ^2^University of Paris Nanterre, Hospital Cochin Paris, Paris, France; ^3^University of Paris, Hospital Cochin AP-HP, Unite Inserm 1018, CESP, Paris, France; ^4^Outpatient Unit for Research, Teaching, and Practice, Faculty of Psychology, University of Vienna, Wien, Austria

**Keywords:** asylum seekers, explanatory models, family, post-traumatic stress, transnationalism, trauma, undocumented migrants

## Abstract

**Context:** Diasporic Cameroonians are increasingly leading a transnational life in which family members are sustained through networks of relations and obligations. However, before arriving in Europe, the vast majority of African migrants who take the Mediterranean route are exposed to trauma and hardship. Moreover, the joint occurrence of forced displacement, trauma, and extended separation from families has a significant impact on mental health.

**Objectives:** This study explores the role of culture-specific conceptualizations of family structures and transnationalism in explanatory models of post-traumatic stress disorder (PTSD) among male Cameroonian asylum-seekers and undocumented migrants in Europe.

**Methods:** An in-depth study of two samples of Cameroonian migrants with a precarious residency status in Europe was conducted. Focus group discussions and interviews were carried out with asylum seekers in Germany (*n* = 8) and undocumented migrants and failed asylum seekers in France (*n* = 9). The verbatim transcripts of these interviews served as the data for interpretative phenomenological analyses.

**Results, Analysis, and Discussion:** Family was conceptualized in religious and spiritual terms, and relational spirituality appeared to be a crucial element of family cohesion. Explanatory models of PTSD were mainly based on an intersection of family and spirituality. The disrespect of transgenerational, traditional, and spiritual obligations toward parents and ancestral spirits represented a crucial causal attribution for post-traumatic symptoms.

**Conclusions:** Conceptualizations of post-traumatic stress were based on a collective family and spiritual level instead of an individualized illness-centered perception. The Western psychological and psychiatric perspective on post-traumatic stress might conflict with traditional, religious, and spiritual practices in the context of family conceptualizations of Cameroonian forced migrants with a precarious residency status.

## Introduction

According to the United Nations, the security and human rights situation in Cameroon has significantly deteriorated (1), leading to substantial displacement and irregular migration flows toward the European continent via the Mediterranean route (1–3). Further perpetuating the high rates of irregular migration is the difficult economic situation of Cameroon, with families' incomes declining, the costs of education rising, and the unemployment rate growing (4). France and Germany are the top two destinations of resettlement for Cameroonian migrants in Europe, as the countries are bound by cultural, historical, and post-colonial ties (2, 4, 5). Many forced migrants need to reorganize relationships within the fragmented family, redefine the roles and relationships within the changed family structure, adjust to a new environment, and reevaluate future perspectives (6). Accordingly, the term “transnationalism” describes the unique diasporic experiences of migrant families in terms of maintaining family relationships within and across nations simultaneously (7). Diasporic Cameroonians are increasingly leading a transnational life, in which family ties are sustained through networks of relations, obligations and resources that are located in different nation states (4, 8). The complexity of relationships that arise from transnational connections calls dominant discourses about family bonds into question and requires the adoption of new theory and treatment considerations within a transcultural context (9, 10). Transnational families are one form of contemporary families that demand new analytical frameworks for understanding family relationships (10).

Prior to their arrival in Europe, the vast majority of African migrants taking the Mediterranean route have experienced traumatizing events that have altered their lives drastically (11, 12). During their journey, migrants often depend on human traffickers and are liable to experience physical and sexual violence, torture, and abduction (11, 13–15). When a person experiences a trauma, the range of psychiatric consequences can, inter alia, include anxiety and depressive disorders (16). Moreover, lasting impressions can lead to health problems that are labeled as post-traumatic stress disorder (PTSD) (17). The concepts of trauma and PTSD have been addressed through the lens of an individualistic Western medical nosology (17). According to the World Health Organization (18), PTSD is defined by the core symptoms of intrusiveness or re-experiencing the trauma (nightmares, flashbacks, and recurring memories), hyperarousal (difficulty sleeping, irritability, and hypervigilance), and avoidance (reminders of events and dissociation) (17). Past research in asylum-seeking and refugee populations from Africa found PTSD rates of up to 79% (19–21). Individuals without secure residency status—be it pending cases (22, 23), rejected asylum seekers (24, 25), or undocumented migrants (26)—appear to be at a particularly high risk for ongoing mental health problems and post-migration stress (25, 27).

In Cameroonian cultures, extended family systems and strong kin relations are important, since they provide a sense of belonging and solidarity (4). However, many individuals, traumatized by the hardship endured during their migration path, face a situation of radical rupture and separation from their families remaining at home (28, 29). Accordingly, past research suggests that asylum seekers and forced migrants have the impression of being ripped out of their familial environment, which might provoke feelings of distance, exclusion, loss, and grief (15, 28, 30). Moreover, leaving behind families living in poverty, privation, and danger may precipitate and perpetuate the development of symptoms of PTSD (7, 29, 31).

Critics have blamed the one-sidedness and reductive character of a purely symptom-oriented conception of trauma and post-traumatic stress (32), and have noted that PTSD may be an overly narrow characterization of traumatic stress across different cultures (17, 32–34). Taking individuals' sociocultural backgrounds into account, explanatory models describe the way in which individuals perceive, interpret, and respond to illness, and demonstrate how causality and help seeking behavior vary across cultures (35–39). The concept of explanatory models was first introduced by the medical anthropologist and psychiatrist (40). Explanatory models are shaped by the underlying socio-cultural contexts and can be understood as fluid and multilayered constructs that reflect the cultural knowledge of each individual (37, 41). Conceptualizations about health are, therefore, cultural. They vary widely across societies and sociodemographic groups and should not merely be defined by measures of clinical care and disease (42). In this regard, symptoms of mental disorders and explanatory models are two different constructs and it is possible that there may be different explanatory models for the same symptoms. Whereas, members of white middle-class communities in Western cultures may be apt to view PTSD as a medico-psychological problem requiring individual examination and professional treatment, members of African immigrant populations may conceptualize the symptoms as social problems, requiring collective and relational forms of treatment (15, 38, 43). Considering the important role of the extended family system in Cameroonian society, explanatory models of PTSD in this group cannot be assessed without reference to the family and its involvement (4). Furthermore, to fully understand individual psychopathology, culture specific and transnational conceptualizations of family structures and cohesion need to be taken into account (44). Therefore, researchers and clinicians need to consider not only patients' culturally shaped explanatory models, but also their culture-specific and transnational family conceptualization, as these most commonly differ from Western nuclear family norms (45).

In the literature on African causal theories for mental disorders, the role of family and spiritual etiology is often emphasized (46–48), a finding that also emerged in asylum-seeking populations from Sub-Saharan Africa in Western resettlement countries (49). Past research has documented attributions to causes such as dead, missing, or separated family members; and spirit possession or curses (50). Rather than seek help from a clinician, migrants preferred to first use family support, prayer, or traditional therapies (50, 51). In this regard, scholars have stated that spiritual beliefs and practices are tightly interwoven with sociocultural influences, and vary greatly across and within cultural groups (52). Moreover, spirituality is deeply entrenched in most aspects of family life, influencing the ways in which families deal with adversity, their experience of suffering, and the meaning of symptoms (53). In previous work, we described the interrelatedness of family and spirituality and the way in which symptoms of PTSD were conceptualized among asylum seekers from Sub-Saharan Africa in Germany (15, 43). We found that perceptions of PTSD were conceptualized, inter alia, by familial discords intersecting with supernatural and spiritual levels. Moreover, the role of the family was emphasized with regard to the management and treatment of symptoms of PTSD (43). Thus, we assume that culture-specific conceptualizations of family structures and a relational form of spirituality might influence explanatory models of PTSD of Cameroonian migrants. To date, however, there is no research examining the role of family in explanatory models of PTSD in this group. Moreover, most evidence on asylum-seeking and migrant populations still conceptualizes PTSD primarily on an individual illness-centered level, with the cultural and social embeddedness remaining relatively unexplored.

### Theoretical Framework and Study Objectives

The objective of the present study was to explore lay representations and explanatory models of PTSD in Cameroonian migrants with a precarious residency status in Europe, who had migrated via the Mediterranean route. Moreover, in the present paper we focus on the role of culture-specific conceptualizations of family and transnationalism in explanatory models of PTSD. As analyses have shown that African migrants coming to Europe typically show the characteristics of being young, male, and optimistic about attaining a higher standard of living (54), we focused on a male sample in the present study. As study locations, we chose France and Germany, as these are the top two destinations of resettlement for Cameroonian migrants in Europe.

We followed a combined emic–etic research approach by focusing on the etically defined construct of PTSD and employing emic methodologies to measure it. Through such an approach, an emically defined etic construct can be obtained, which facilitates comparisons across different cultural and demographic groups (53). Accordingly, the present research used qualitative methodology that allowed us to focus on the intrinsic cultural and demographic distinctions that are meaningful to the young Cameroonian migrants regarding the intersection of culture-specific family conceptualizations on the one hand and symptoms of PTSD on the other hand. Moreover, the present study is placed within an illness explanatory model framework using a vignette methodology (35, 54, 55). In this regard, we aimed to explore culturally shared and constructed perspectives regarding explanatory models and illness representations of forced Cameroonian migrants, by extending the explanatory model approach beyond just the patient's perspective [see also (38, 55, 56)]. Rather than focusing on a clinical population, we included lay people of Cameroonian origin in the present study. The high prevalence of PTSD among asylum seekers of Sub-Saharan African origin suggests that a large proportion either experiences this disorder themselves or has to cope with other members of their families or communities who experienced trauma and suffer from traumatic stress (55). Research suggests that, particularly in communal cultures, family members have a strong influence on representations of mental health and illness (57). Thus, the views of lay people may be highly informative about how forced migrants with a precarious residency status conceptualize symptoms of PTSD.

In the present study, we conceptualize culture not solely as a set of habits and beliefs about perceived health and illness, but also as political, economic, legal, ethical, and moral practices and values (42). In order to gain a more comprehensive understanding about the explanatory models of PTSD in Cameroonian forced migrants, we aimed to consider the intersectionality of cultural explanatory models and illness representations on the one hand and the current legal and economic situation on the other hand. As common practice in cross-cultural research, we focus on culture at the level of nationality, while recognizing that nation states are rarely homogeneous (55). However, scholars have characterized Sub-Saharan cultures by an emphasis on embeddedness and hierarchy that implies expectancies of obedience, conformity, and group identification (58). We therefore assume that Cameroonian forced migrants might adhere strongly to culturally shaped representations and beliefs as regards how they understand symptoms of PTSD (55). On the other hand, we focus on culture at the level of a particular sociodemographic, legal, and economic level, as the precarious living situation of forced migrants on a foreign continent might have a strong influence on explanatory models as well. The present study therefore contributes to a dialogue between medical anthropology, sociology, social psychology, and transcultural psychiatry.

## Procedure

The study involved discussions with 17 young men from the French-speaking part of Cameroon who had taken the Mediterranean route in order to seek refuge in Europe. The first author FG undertook individual research interviews and focus group discussions with eight Cameroonian migrants applying for asylum in Germany, and nine undocumented migrants and failed asylum seekers (who had no authorized or legally recognized presence) in France.

Ethical approval was obtained from the local review board of the Department of Psychology, University of Marburg, Germany, and all participants provided informed consent prior to participation. Contacting the participants required anthropological work by the first author FG. Purposive sampling was utilized in order to approach participants during ethnographic fieldwork in suburban areas of Paris and urban areas of Southern Germany, where asylum seekers are commonly located in accommodation facilities (59–61). This gave the researcher the opportunity to immerse herself into her study field (60). Moreover, it also enabled us to study the research topic in the actual living environment of the participants, in order to get a feel of the life world of migrants with a precarious residency status in France and Germany (60). Concurrently, snowball sampling was employed to recruit additional participants. In this case, we asked men to nominate friends or acquaintances similar to themselves. Specifically, we asked to suggest other Cameroonians with a precarious residency status, who had taken the Mediterranean route in order to come to Europe. Though the snowball method obviously calls into question the representativeness of the sample, the authors' own previous research experiences within African asylum seeking migrant communities, as well as that of other researchers (38), showed that it is necessary for this particular population. Especially with regard to the privacy concerns among undocumented migrants, alternative sampling strategies are unlikely to be equally successful [see also (38)].

For the study, men had to self-identify as having migrated from Cameroon via the Mediterranean route, having a precarious residency status (being undocumented/seeking asylum/failed asylum), and aged 18 years or older. The research interviews and focus group discussions took place in the spring of 2017 in Germany and the summer of 2017 in France. In the present study, no specific assignment of participants to individual interviews or focus groups was made. Participants rather naturally allocated themselves to the different survey methods. We applied this strategy of data triangulation to combine qualitative data drawn from different sources that complement each other in order to gain a richer understanding of the phenomenon under research [see also (62)]. Through such an approach, we ensured a contextual basis for making culturally and demographically sensitive interpretations [see also (63)].

Most of the interviewees expressed concerns about being interviewed in an official facility such as a university. In France, we therefore decided to conduct the discussions in a quiet park that seemed appropriate. The focus group discussion undertaken in Germany took part in prepared rooms in participants' accommodation facilities. The interviews and focus group discussions lasted between 30 and 90 min and were undertaken in French. The mean duration was 1 h. Each interview was audio-recorded and transcribed for later analysis.

We asked participants to provide demographic information and gave them a standardized vignette, illustrating a hypothetical close friend describing symptoms of PTSD according to criteria outlined in the International Classification of Disease, 10th revision (18, 64). Neither the terms trauma nor PTSD were used (see Table 1). Participants were asked to imagine the scenario and to indicate their ideas, concepts and impressions concerning the described condition, using the Short Explanatory Model Interview [SEMI; see Table 2 (65)]. The SEMI is a semi-structured interview using open-ended questions to elicit explanatory models, exploring the respondent's cultural background, the nature of the presenting problem, and help-seeking behavior. The first author FG encouraged participants to talk openly about their attitudes and experiences with the aim of eliciting the concepts held, and to explore their relationship to the current situation and culture. Probes were employed to confirm the concepts mentioned and to explore areas of interest that were not raised spontaneously (65). Participants could discontinue the focus group discussion and interviews at any time.

**Table 1:** The vignette.

“Since the flight, I have become a totally different person. In the evenings, I lie in bed and then these thoughts and images come and I lie awake forever. Now I have reached a point where I realize I can't go on like this anymore … Sometimes I scream at night and I wake up drenched in sweat because of the nightmares. If I have arrived somewhere and there is a noise, I wince. There it is again. I can't turn it off, it's like an electric shock that immediately goes straight up and triggers intense sweating. My wife/My husband accuses me of often being aggressive, easily irritable and she/he is afraid of my outbursts of rage. That's why I prefer to withdraw myself because I always have a feeling that no one can be trusted anymore. Many things just don't interest me anymore. Sometimes my environment appears distant and unreal and I have a feeling of “standing next to myself,” then I become totally numb. Afterwards I sometimes can't remember what has happened. I have no hope left anymore…”

**Table 2:** Interview guide with regard to a case vignette in which a hypothetical friend described symptoms of post-traumatic stress disorder.

•What, if anything, is the problem? •What do you call these problems? •Does he or she have an illness? If yes, what is it? •Do you have an understanding of this discomfort? •What are the causes of his/her problems? •How can you explain what this person is going through? •What should he/she do about it? •Has he/she the power to influence the discomfort? •Where would he/she seek help from? •What should a practitioner do about it? •What could a treatment look like?

*We formulated the questions based on the Short Explanatory Model Interview [SEMI, (65)]. All questions were open to further exploration*.

### Data Analysis

The analyses presented here are part of a larger study investigating illness representations for PTSD in asylum seekers from Sub-Saharan Africa (15, 43). Interviews and focus group discussions were audio-recorded and transcribed verbatim before being subjected to Interpretative Phenomenological Analysis [IPA; (66)]. We used the analysis software MAXQDA version 12 to organize and manage the data. IPA is one of the best known and most commonly used qualitative methodological approaches in psychological research (67). It is concerned with the detailed examination of personal lived experience, the meaning of experience to individuals, and how individuals make sense of that experience (67). The present study applied an inductive research methodology aiming to build a theory based on the collection of empirical data rather than to validate or invalidate an initial hypothesis (68). A detailed analytic treatment of each case was followed by the search for patterns across cases, resulting in the presentation of shared themes and particular ways in which these themes played out for individuals (67).

Following different stages described by Smith and Shinebourne (66), the first author began by summarizing and connecting statements recorded for each piece of respective discourse after closely reading the first transcript several times. Initial notes and responses to the material were captured and translated into preliminary themes at one higher level of abstraction. Several themes were interlinked and subthemes were developed, which were visualized in the form of a heuristic chart. This procedure was repeated for each transcript and patterns were established cross-case. This resulted in a master table of themes for all of the transcripts. In a first step, this process was undertaken separately for the two groups of participants (divided by country of residence and residence status), resulting in the presentation of shared themes in a first place separately. In a second step, the themes and subthemes found separately for the two groups were compared with each other. Within this process, we found no differences with regard to explanatory models and family conceptualizations between migrants with different residency statuses (asylum-seeking or undocumented) or regarding the country of residence (France or Germany). For this reason, we treated them as one group within the present study.

Furthermore, the lead author FG received feedback from Cameroonian psychologists and psychiatrists, and the audited themes were reviewed and discussed with other Cameroonian migrants to ensure that conclusions were culturally accurate and well-derived from the transcripts (66, 69).

### Sample

Five qualitative interviews and four focus group discussions were completed, with a total of 17 participants. In France, nine young men participated in the study within two focus group discussions and five interviews. In Germany, eight young men participated in two focus group discussions. Overall, the 17 participants had an age range from 20 to 30 years, with a mean age of 26 years. Participants in France had a mean age of 26 years and participants in Germany 25 years. Most of the participants were single, and seven participants had children. One participant was married. Five participants were without formal education, one participant had obtained a school leaving certificate, two a secondary school certificate, and seven had a higher-education entrance-level qualification. Participants' mean duration of stay in France was 1 year and 9 months (range 3 weeks−5 years), whereas the mean duration for participants in Germany was 1 year and 6 months (range 1–2 years). Most of the participants were Christians (12 participants), three participants stated not being religious, one participant was Muslim, and one participant was Animist. Participants were from various ethnic backgrounds, with most identifying themselves as belonging to the group of the Bamiléké (seven participants), the largest ethnic group in Cameroon. Other participants identified themselves as belonging to the group of the Bassa (four participants), the Sawa (three participants), the Foulbé (two participants), and the Beti (one participant).

## Results and Analyses

Participants responded promptly to the vignette, and all participants seemed to find it interpretable. Moreover, they stated that the described PTSD symptoms appeared familiar, as they identified them in either themselves or in somebody they knew. The initial objective of the research project was to explore explanatory models of symptoms of PTSD in lay Cameroonian forced migrants who had taken the Mediterranean route to Europe. The importance of family, spirituality and transnationalism in explanatory models appeared as in response to our initial research question and emerged in the themes generated from the transcripts (Table 3). The relevance of family in explanatory models of symptoms of PTSD was strongly emphasized in all of the discussions, with the majority of participants bringing up the topic of their own volition.

**Table 3:** Structured presentation of the themes and subthemes of the interpretative phenomenological analysis.

**Superordinate themes**	**Subthemes**
Conceptualizations of family	Extended self
	Transcendental family cohesion
	Religion
	Intergenerational obligations
	Tradition and customs
Family in the context of forced migration	Assignment of a family mandate
	Challenges for families
Perceived causes for symptoms of PTSD	Incapacity to adhere to intergenerational obligations
	Malediction
	Disrespect of parents/elders
	Disrespect of ancestors
	Failure to carry out cultural customs
	Incapacity to support one's family remaining at home
Sources of resilience	Reliance on family network
	Receiving blessings from parents
	Performance of cultural customs
	Reliance on ancestors

### Relational Spirituality and Conceptualization of Family Cohesion

In general, participants described family in the Cameroonian context as conceptualized not only biologically, i.e., including parents, siblings, grandparents, uncles, and aunts, but also socially and geographically, i.e., including the parents' village communities. The village of origin appeared to be of special importance in creating a sense of belonging and rootedness in a traditional family context. The young men strongly perceived themselves to be members of these collectives and referred to their social positions within their families and their village communities. They often made use of their collective self and their membership within a social collective by referring to “we” instead of “I” when expressing their opinion. In particular, the young men emphasized the special importance of their parents within the familial framework. This was described as being embedded within a spiritual and religious context.

“In the Bible it is stated: After God there is the parents. (…) So to speak your mother (…) and your father are like God too. That's why we are trying to make an effort to be obedient”.

Participants conceptualized religion and spirituality in relational and familial terms, and relational spirituality appeared to be a crucial element of family cohesion within the Cameroonian family, especially characterizing the relationship with the parents and elders. Scholars have defined relational spirituality as the way in which particular aspects of spirituality affect the formation, maintenance, and transformation of family relationships (70–72). This spiritual conceptualization of family cohesion even included transcendental connections with deceased parents, grandparents, ancestors, and ancestral spirits. The communication was described as occuring during sleep *via* dreams and apparitions, often mediated by the parents.

“Where we come from this will mean that the elderly are not dead. The ancestors/”

### Challenges for Families in the Context of Forced Migration and Transnationalism

In the context of migration, the conceptualizations of family were described as entailing obligations and responsibilities, especially toward the parents. The main reason behind migration was reported to be implicit or explicit assignments of a family mandate in order to improve the family's socio-economic situation in Cameroon. In this regard, participants described holding the main responsibility to financially provide for the family in Cameroon.

“If you are leaving your country for Europe, it's for one purpose: Seeking help for your family. (…) It's the full faith of a family. There is a family waiting for you. You have to help them. (…) So if you are combating, it's for your family”.

In this regard, participants perceived their migration as a complex and multifaceted challenge and a combat on behalf of their families. The migration path via the Mediterranean route was described as especially burdensome for the migrants' families. When reporting about the hardships encountered during their traumatizing journey, participants frequently chose their family's rather than their own perspective. They outlined that for their families, their migration meant not only separation and loss of contact, but also uncertainty about their physical integrity and survival. The participants reported abductions by human traffickers (“*coupeurs de route*”) and the extortion of their families in Cameroon as common occurrences during their journey. Some participants disclosed that their families were obliged to sell their assets in order to free their kidnapped sons.

“When you are walking in the desert (…) There are these *coupeurs de route* (human traffickers). (…) They catch us and make us all kneel. They search us. (…) They see that you don't have any money: if they are kind they will let you off. If they are not kind, they will just kill you. They break your legs. (…) They kidnap you. When they kidnap you, they say: those who have money pass. Those who don't have money stay here. (…) And when he finishes like that he will kill you. They will ditch your corpses everywhere”.
“They take Africans to make business, human trafficking. Tomorrow they call your poor family, your family that has nothing to eat, to send money. Your family sells the house that they are living in. (…) There are families that no longer have a house, they have been left homeless. They have sold everything to help their son on his journey”.
“There was no money left. We lost all of the assets we had. What little we had, we took it to pay my journey. I was abducted three times. I lost 3000 Euro during my journey. As I left for my adventure, I did not foresee this. We had to sell our land”.

### The Role of Family in Explanatory Models of Symptoms of PTSD

The conceptualization of symptoms of PTSD on a relational, familial, and spiritual level appeared to be an important part of explanatory models of the Cameroonian men. Transgenerational and spiritual obligations toward parents and ancestors, and the deliberate disrespect or the perceived incapacity to adhere to these obligations were construed as a crucial causal factor for symptoms of PTSD. Independently, all of the participants brought up the concept of malediction (*malédiction*) for explaining the symptoms of PTSD. Embedded within a spiritual and religious context, a malediction was described as being associated with disobedience to the parents and the disregard of the parents' requests. A malediction was explained as originating from disrespectfully treated or ignored parents and as having the potential to cause serious misfortune in the future life. The transmission was believed to happen from the parent to the child through words:

“Actually, it's like somebody cursed her. Because she does not recognize herself anymore. It's like somebody cursed her with a malediction”.
“Your mother may be very angry, very. She curses you. (…) Everything you are going to do won't work. You are blocked. (…) Imagine now that with her anger (…) she dies. Your mother dies being angry with you. (…) This is a malediction”.
“For me, the only thing I believe in is the word. For me, words have a significant effect. It means for example, one parent who is dissatisfied with you, employs certain words. This can influence your life. Like the Bible says before: there was the word. This means that God gave a power to the word. (…) So if there is a conflict in your family. Maybe you were disrespectful to an elder or a father or your grandfather, he can curse you. (…) A malediction comes from the word. (…) He can speak some serious words that have aftereffects in your life”.

Participants reported the disrespect of ancestors and a failure to honor ancestral spirits as another important cause for symptoms of PTSD. This could be provoked by not honoring the family's or the parents' estate in disregarding their directives. Moreover, another causal attribution for upsetting ancestral spirits was the failure to carry out distinct cultural customs and traditions, such as sacrificing food to the ancestors' tombstones.

“I am giving you a short example: You are very rich. You have a lot of money. (…) But you have forgotten your great grandfathers. (…) They see that you have forgotten about them. They will make you poor until you have nothing left anymore. (…) This is a malediction. And this can also show in symptoms like that”.
“It's always the customs. You have to renew the tombstones and often buy oil to blend it with salt and everything. You blend it and put it onto the tombstones. (…)You will be asked what you have; you eat with your ancestors. Yes, don't be stingy. It's not good. Because if you don't do it, you can be cursed by the ancestors”.

In the context of migration, the young men felt separated from their native land and described that in some cases they were incapable of carrying out certain cultural customs and traditions regarding their ancestors. Moreover, some felt that their spiritual connection was interrupted by the Mediterranean Sea and the hardships encountered on their journey.

“Before, when I was in Africa, things came to my mind. But since I am in Europe these things don't come to my mind anymore. But in Africa I saw while I was asleep. (…) My ancestors talked to me the day before, while I was sleeping to tell me what would happen the next day. (…) I crossed the Mediterranean Sea, because the Mediterranean Sea is a sacred water. (…) It remained in Africa”.

As another important cause of the described symptoms, participants stated the perceived pressure regarding their financial familial obligations and their own incapacity to support their families remaining at home. Beforehand, these implicit or explicit family-instructed assignments were often reinforced by unrealistic and paradise-like ideas about the life circumstances in Europe. Upon finally arriving in Europe, the participants described their shattered assumptions about the realities of life and the dilemma between carrying high expectations and being bound to their transgenerational obligations on the one hand and their own precarious situations on the other hand. At worst, participants described that this could lead to a complete separation from one's family, causing trauma and symptoms of PTSD.

“If you leave your country in order to get to Europe, it's for one objective: Helping your family. (…) You see somebody who is so eager to go to Europe. He knows in Europe he will find the diamond, gold. But afterwards, he does not find the diamond. (…) He arrives in Europe and he understands that it's not how he has imagined”.
“You know that you have nothing. (…) It's better to part. You see the way you settle down. You know that you have nothing to give”.
“It's a lot. He does not even know his family any more. Since I lived with him, I have never heard him calling his family. So, he lost everything. (…) Actually, I say this may cause a disorder like this”.

### The Role of Family in Conceptualizations of Cure and Resilience

In general, the cure was strongly dependent on the perceived cause of the symptoms. Most commonly, the treatment decisions were placed in a familial context and help-seeking was reported to rely strongly on the parents or elders. These were stated as the first point of orientation, especially within a spiritual and religious context. Psychological problems were described as being resolved within a family network, and parents and elders were reported to give advice and guidance in the case of mental distress.

“You don't have the power yourself. It's the mother who will take you somewhere. (…) Maybe she goes to see a seer for this. (…) Either she will say: My son you need to pray. You always have to pray. Or go and see our ancestors and ask them what is wrong. Why does my son have these problems?”

Most commonly, resilience and cure for mental distress was understood to be embedded within a familial context highly intertwined with spiritual, religious and traditional practices. If the cause was perceived to be upset ancestral spirits, the performance of sacrifices was reported as an effective cure to pacify the ancestors and reduce symptoms of mental distress, most commonly mediated by the parents. In this case distinct cultural customs need to be carried out, such as sacrificing food to the family or the ancestors' tombstones, in particular goat meat, red palm oil, salt, and rice. If these practices cannot be carried out by the affected person directly, the sacrificial offering could be performed by the affected person's family in the country of origin (*appeler au pays*).

“Maybe you are trying to do something but it is not moving forward (…). You approach your mother: Mother, I am trying something here but something is always blocking. (…) Your mother tells you the next day that the ancestors could be upset with you. And if your ancestors are upset, (…) go and see your ancestors. You speak to them. You nourish them. They need to eat as well”.

At the same time, the ancestors were described as being a great source of resilience and protection against every form of harm and mental distress.

“Because after God it's the ancestors who are protecting you. You have to honor your ancestors. (…) They can protect you against witchcraft or against those who want to hurt you, who want to kill you, who want to harm you. (…) One of your ancestors came and gave my mother a sign: your mother has nightmares about you. (…) Somebody wants to harm you. As soon as she has these nightmares, your ancestors will tell [her] what to do because your child is in danger. It's happening like that, where we come from”.

In the context of migration, the migrants' family appeared to be the main root of resilience while coping with the hardships encountered during their traumatizing journey. Participants independently brought up the concept of receiving blessings (*la bénédiction*) from parents and the family elders as a crucial source of resilience during their journey and in life in general, and as a cure for the symptoms of PTSD and mental distress. In order to receive a parent's blessing the child needs to respect the transgenerational obligations and duties, and carry out the traditional spiritual customs.

“Before you are leaving, we make a family reunion. It's a reunion managed by the family of the person that will leave and his whole family needs to come. That ensures that everybody blesses him in order to protect him. (…) They will do a little ceremony”
“The motivation of my family. They were motivating me a lot. You can! I arrived!”

## Discussion

The use of an explanatory model approach allowed a detailed exploration of the role of culture-specific conceptualizations of family in concepts of PTSD in lay male Cameroonian migrants with a precarious residency status in Europe, who took the Mediterranean route. Moreover, it enabled us to gain an understanding of the impact of forced migration and transnationalism on relational patterns and family cohesion.

The present results show that Cameroonian forced migrants with a precarious residency status in Europe understood family not only in biological, but also in spiritual and religious terms. Thus, religion and spirituality was conceptualized in relational and familial terms, and relational spirituality appeared to be a crucial element of family cohesion, especially characterizing the relationship with the parents. Overall, studies in different populations suggest benefits of religion and relational spirituality for family functioning and the quality and stability of relationships (52, 73). However, research to date is still limited, and previous studies have predominantly been conducted within Western, highly religious, white Christian families (52). This spiritual conceptualization of family cohesion of Cameroonian migrants with a precarious residency status even included transcendental connections with ancestral spirits. In general, past research has found that transcendent values foster healthy family functioning (52). Moreover, a shared belief system that transcends the limits of family members' experience enables better acceptance of inevitable risks and losses (52). Within a transcultural perspective, the present findings emphasize the intersection of family and spirituality in Cameroonian migrants with a precarious residency status and propose a corrective to a mainly Westernized, overly individualistic and distinct understanding of spirituality and family (70).

Regarding the impact of forced migration and transnationalism on relational patterns and family cohesion, the present study revealed that migration appears to be a multifaceted challenge for families. The migration path via the Mediterranean route was described as especially burdensome, and migration meant not only separation and loss, but also uncertainty about the physical integrity and survival of the departing family member. Therefore, family separation in asylum seekers and forced migrants can be understood as a transnational relational stress and ambiguous loss, insofar as the temporary absence of other family members cannot be fully acknowledged due to the perpetual uncertainty and permanent risk to them (29). Against this background, current European policies that intend to restrict irregular immigration and hamper asylum procedures perpetuate the negative consequences of separating families even more, by impeding reunification (9, 10).

With regard to explanatory models, Cameroonian migrants with a precarious residency status conceptualized symptoms of PTSD on a relational, familial, and spiritual level. The transgenerational and spiritual obligations toward parents and ancestors and the deliberate disrespect in failing to adhere to these obligations were construed as crucial causal attributions. Within this context, the concept of malediction (*malédiction*) appeared to be an important cause of the symptoms. Moreover, the disrespect of ancestors and failure to honor ancestral spirits was another important cause for symptoms of PTSD. These findings are largely in line with past research on explanatory models of mental illness in other conflict-affected African communities and asylum-seeking populations from Sub-Saharan Africa, which documented a failure to honor ancestral spirits and curses to be an important causal attribution (46, 50). Another important causal factor in explanatory models of PTSD was the enormous social pressure to adhere to implicit and explicit family assignments and obligations to send remittances to serve the family interests. The dilemma between the migrants' precarious situations in Europe and the high expectations was reported to be an important source of mental distress, which could lead to complete separations from families. This finding should alert therapists to develop a critical awareness of migrants' transnational family contexts and the tremendous social pressures and obligations inherent therein (7).

Furthermore, Cameroonian migrants with a precarious residency status placed resilience and cure for trauma and PTSD within a family context highly intertwined with spiritual, religious, and traditional practices, and receiving blessings (*la bénédiction*) from parents and the family elders was stated as the most important source of resilience. In this regard, abundant research documents the powerful influence of spiritual beliefs and practices for recovery and resilience (52, 74), and the importance of family cohesion has been described as a crucial element of resilience and recovery in immigrant and refugee populations (75–78). The religious and spiritual conceptualization of family relations in Cameroonian migrants provides a strong sense of cohesion and sense of belonging. Members are able to perceive the experience of forced migration, however painful and uncertain, from a broader perspective that creates a sense of events and fosters hope (52). However, still little is known about what happens to the family remaining in the country of origin. Future research might engage in binational studies in order to assess conceptualizations and perspectives of those members of the same family who stay behind (9). Moreover, it might be interesting to investigate lay representations and explanatory models of trauma and PTSD, as well as family conceptualizations among migrants with a regulated residence status in order to further explore the influences of the precarious living situation.

The present results demonstrate that on the one hand family is experienced as supportive in Cameroonian migrants with a precarious residency and can promote resilience. On the other hand, family can be perceived as a burden due to the feeling of responsibility toward ancestors or feelings of guilt toward family members who have stayed behind. In future quantitative research, it might be interesting to explore individual differences linked to own stressful experiences among a larger group of participants. Moreover, it might be interesting to explore whether subjective explanatory models open scope for a specific way of coping, such as praying or creating individual meaning, in contrast to the peritraumatic and often re-enacted powerlessness and helplessness, which are frequent symptoms in treatment.

Our results suggest that the Western psychiatric perspective on post-traumatic stress and the individual patient-centered approach employed by Western-trained psychotherapists might conflict with traditional, religious, and spiritual practices in the context of family conceptualizations of Cameroonian forced migrants with a precarious residency status in Europe. Accordingly, an increased awareness among healthcare providers of how family context and the intersection with religious and spiritual belief systems impact the mental health of migrants is needed (26). Clinicians might respectfully inquire about the meaning and importance of religion and spiritual beliefs and practices in the individual and family life, explore spiritual and family sources of distress, and identify spiritual and religious resources (52). However, many Western therapists feel that their training leaves them ill-equipped in this regard and find it uncomfortable to address transcultural, religious, and spiritual topics (52, 79). Thus, our results uncover the need for culturally relevant tools for screening and addressing the mental health needs of patients from different cultural backgrounds. However, on the one hand scholars have criticized that the assessment of explanatory models has not yet been integrated into routine clinical practice, regardless of its importance for the improvement of cultural competency in assessment, diagnostic validity, and the therapeutic relationship (35, 80). Moreover, the majority of published guidelines on cultural and spiritual assessment and interventions are designed predominantly for individuals, and fail to differentiate between individual and family spirituality (81). On the other hand however, it should be noted that in view of the frequency of trauma-related disorders in asylum seekers and forced migrants, this demand might often represent an excessive requirement for clinicians and psychiatric institutions. Therefore, clinicians should adopt an attitude of cultural humility (82, 83), were practitioners have an interpersonal stance that is other-oriented rather than self-focused, characterized by respect and lack of superiority toward a patient's socioeconomic and cultural background (83). In this regard, clinicians should show interest in the patients' assumptions about their illness and see themselves as non-paternalistic learners. In this way, distrust can be reduced, misunderstandings can be prevented and a discourse creating coherence on different disease concepts can arise without running risk of cultural stereotyping.

## Limitations

While interpreting the present results, some limitations and methodological considerations should be taken into account:

In the present study, we combined data of two samples of Cameroonian migrants (asylum-seeking vs. undocumented) residing in two different European countries (Germany vs. France). While we did not find any differences with regard to the conceptualizations of family and its role in explanatory models that could be traced back to the migrants' legal status or their country of residence, an influence of these two factors cannot be fully ruled out. Although there are striking similarities in the relationship of the two host countries with Cameroon (the colonization of Cameroon first by Germany and subsequently by France), there appear to be some major differences as well (e.g., in terms of the extent of the colonization and the decolonization process that followed).

Moreover, we used different qualitative methodologies for the data collection, combining data generated from interviews and focus group discussions. While we did not find any differences with regard to the conceptualizations of family and its role in explanatory models within the data, critics have argued on the one hand that data yielded by focus group discussions are often influenced by social dynamics and frequently describe what people assume they should think, rather than what they actually think (46). However, on the other hand, past research has pointed out that compared to individual interviews, several types of sensitive and personal disclosures were more likely to be reported in a focus group setting, and that some sensitive themes only occurred in the focus group context (84).

We used a general population approach by applying a case vignette design. A disadvantage of this study design is that information about conceptualization of traumatic stress is taken from a third-person perspective. While widely used in transcultural research, vignette methodology is limited in its generalizability to real clinical situations [see also (57)]. Moreover, it is important to point out that not all described symptoms are specific to a diagnosis of PTSD and can also occur in other mental disorders, so that the statements in the case vignette can also be applied to other mental disorders or mental distress in general. Furthermore, we did not particularly assess participants' psychopathological status. Readers should be mindful about the fact that individual psychopathology might shape the interpretation of the presented vignette and even conceptualizations of symptoms of PTSD, whereas severely traumatized persons might differ in their interpretations.

Another important limitation of the present study is the small sample size, which leads to a limited generalizability of the present results. Due to the limited number of participants, caution is warranted in drawing conclusions about the impact of culture on participants' conceptualizations of traumatic stress. Furthermore, we do focus on a particular group of Cameroonian migrants, i.e., those with a precarious residency status who took the Mediterranean route. In general, the aim of qualitative research designs is to investigate a specific phenomenon in a certain population, with a focused locality in a particular context. Therefore, the generalizability of our findings was not an essential attribute (85). Moreover, participants within the present sample highly differed in terms of their educational and socioeconomic background. While analyzing the data, we did not find any particular differences between the participants in this regard. However, we cannot fully rule out an influence of the educational background on conceptions of family and explanatory models of PTSD. Thus, our results represent an important contribution to the present understanding of conceptualizations and explanatory models of symptoms of PTSD, but are only paving the way for future research including larger sample sizes, clinical populations, Cameroonian migrants with a regulated residency status as well as native comparison groups.

Furthermore, data collection and moderation of interviews and focus group discussions were conducted by a white, female researcher, which might have led to a selection bias. Some men might have been reticent to share their opinion due to a feeling of social desirability, or due to differences in gender, social class, and cultural background.

As our sample consisted of only male participants, the present findings represent a male point of view. Thus, an influence of gender on the conceptualization of family and its role on explanatory models of trauma and PTSD can be assumed. This might be of special importance, as the Cameroonian society is highly patriarchally organized, and women might have very different perspectives on family structures and transgenerational obligations. Therefore, future studies may wish to consider investigating female samples in particular.

Even though we focused on migrants from Cameroon, we recognize that countries are rarely homogeneous societies with unified cultures (58). As such, it should be kept in mind that Cameroon is one of the most diverse countries in Sub-Saharan Africa in terms of geography, language, ethnicity, and religious affiliation (4). While we took the ethnic, religious, and geographical diversity of participants into account when analyzing the data, particular conceptualizations of family and its role in explanatory models might still vary between different cultural and ethnic groups of Cameroon.

## Conclusion

The conceptualizations of trauma in Cameroonian forced migrants with a precarious residency status were based on a collective family, religious, and spiritual level instead of an individualized illness-centered perspective, challenging the narrow conceptual framework of PTSD. Thus, the Western psychological and medical perspective on traumatic stress might conflict with traditional, religious, and spiritual practices in the context of family conceptualizations of Cameroonian migrants with a precarious residency status. When working with traumatized migrants from Cameroon, health professionals need to use a broad and flexible multifocal lens (6). It is necessary to acknowledge the diversity of belief systems and attitudes within cultures and to attempt to understand each individual context as embedded within a culture-specific family context and a particular demographic situation. Clinicians working with traumatized Cameroonian migrants with a precarious residency status can respectfully address patient's cultural needs by assessing the family history and exploring the intersection with religion and spirituality.

European health care systems need to take into account the changing demographics of populations and family configurations, and provide appropriate mental health care for the growing number of forced migrants and migrants with a precarious residency status (3, 14, 86). Moreover, mental health care providers require skills in trauma-informed and transcultural care in order to meet the complex needs of populations from diverse cultural and demographic backgrounds. The training of Western clinicians and health care providers working with migrants from diverse cultural backgrounds should incorporate knowledge about explanatory models and specific cultural, religious, and spiritual characteristics. Furthermore, therapists must pay attention to the complex interactions within family systems and even develop transnational collaborations in order to help their patients (10). Future research might stimulate new theorizing about definitions of family life, how relationships evolve at long distance, and about how to develop systemic and even transnational interventions for separations (10). Finally, family therapists should prioritize the study and treatment of transnational families and consider family members across national borders (10).

## Data Availability Statement

The raw data supporting the conclusions of this article will be made available by the authors, without undue reservation.

## Ethics Statement

The study was reviewed and approved by the local review board of the Department of Psychology, University of Marburg, Germany. The participants provided their written informed consent to participate in this study.

## Author Contributions

FG analyzed and interpreted the data and was a major contributor in writing the manuscript. SS and MM contributed to the interpretation of data and critically revised earlier versions of the manuscript. RM was the senior principal investigator of the study, gave feedback to the analyses and the interpretation of the data, and was a major contributor in writing the manuscript. All authors read and approved the final manuscript.

## Conflict of Interest

The authors declare that the research was conducted in the absence of any commercial or financial relationships that could be construed as a potential conflict of interest.
